# Global birth defects app: An innovative tool for describing and coding congenital anomalies at birth in low resource settings

**DOI:** 10.1002/bdr2.1898

**Published:** 2021-05-05

**Authors:** Helen Dolk, Aminkeng Zawuo Leke, Phil Whitfield, Rebecca Moore, Katy Karnell, Ingeborg Barišić, Linda Barlow-Mosha, Lorenzo D. Botto, Ester Garne, Pilar Guatibonza, Shana Godfred-Cato, Christine M. Halleux, Lewis B. Holmes, Cynthia A. Moore, Ieda Orioli, Neena Raina, Diana Valencia

**Affiliations:** 1 Centre for Maternal, Fetal and Infant Research, Institute for Nursing and Health Research, Ulster University, Newtownabbey, United Kingdom; 2 Biomedical Computing Ltd, East Sussex, United Kingdom; 3 Children's Hospital Zagreb, Centre of Excellence for Reproductive and Regenerative Medicine, Medical School University of Zagreb, Zagreb, Croatia; 4 The Makerere University-John Hopkins University Research Collaboration, Kampala, Uganda; 5 International Center on Birth Defects (ICBD) of the International Clearinghouse for Birth Defects Surveillance and Research (ICBDSR), and Department of Pediatrics, University of Utah, Salt Lake City, Utah; 6 Pediatric Department, Hospital Lillebaelt Kolding, Kolding, Denmark; 7 Latin American Collaborative Study of Congenital Malformations (ECLAMC), Bogotá, Colombia; 8 National Center on Birth Defects and Developmental Disabilities, Centers for Disease Control and Prevention (CDC), Atlanta, Georgia; 9 UNICEF/UNDP/WB/WHO Special Program for Research & Training in Tropical Diseases (TDR), World Health Organization, Geneva, Switzerland; 10 Medical Genetics and Metabolism Unit, MassGeneral Hospital for Children, Boston, Massachusetts; 11 Latin American Collaborative Study of Congenital Malformations (ECLAMC), Rio de Janeiro, Brazil; 12 World Health Organization, Regional Office for South East Asia (WHO SEARO), New Delhi, India

**Keywords:** app, coding, congenital anomaly, global health, surveillance

## Abstract

**Background:**

Surveillance programs in low- and middle-income countries (LMICs) have difficulty in obtaining accurate information about congenital anomalies.

**Methods:**

As part of the ZikaPLAN project, an International Committee developed an app for the description and coding of congenital anomalies that are externally visible at birth, for use in low resource settings. The “basic” version of the app was designed for a basic clinical setting and to overcome language and terminology barriers by providing diagrams and photos, sourced mainly from international Birth Defects Atlases. The “surveillance” version additionally allows recording of limited pseudonymized data relevant to diagnosis, which can be uploaded to a secure server, and downloaded by the surveillance program data center.

**Results:**

The app contains 98 (88 major and 10 minor) externally visible anomalies and 12 syndromes (including congenital Zika syndrome), with definitions and International Classification of Disease v10 -based code. It also contains newborn examination videos and links to further resources. The user taps a region of the body, then selects among a range of images to choose the congenital anomaly that best resembles what they observe, with guidance regarding similar congenital anomalies. The “basic” version of the app has been reviewed by experts and made available on the Apple and Google Play stores. Since its launch in November 2019, it has been downloaded in 39 countries. The "surveillance” version is currently being field-tested.

**Conclusion:**

The global birth defects app is a mHealth tool that can help in developing congenital anomaly surveillance in low resource settings to support prevention and care.

## 1 | INTRODUCTION

Globally, more than 5 million babies are estimated to be born with congenital anomalies (CAs), also called birth defects, each year (James et al., 2018). Ranked as the fourth leading cause of death worldwide for children under five (WHO, 2020a), CAs have been estimated to be responsible for 5–600,000 deaths in 2017, representing 9–11% of all under-five deaths (Dicker et al., 2018; WHO, 2020a). Stillbirths and terminations of pregnancy for fetal anomaly add to this burden of disease (Boyle et al., 2018). Children born with a significant CA who survive have complex medical, social, and educational needs, accounting globally for 48.8 million years of life lost with disability (Roth et al., 2018).

The World Health Organization (WHO) estimates that 94% of all severe CA cases occur in low- and middle-income countries (LMICs) (WHO, 2020b). Many risk factors are more frequent or severe in LMICs, including nutritional deficiencies, maternal diseases, self-medication with potential teratogens, consanguinity, and congenital infections like syphilis, cytomegalovirus, toxoplasmosis, and Zika virus (Kancherla, Oakley, & Brent, 2014; WHO, 2020b). LMIC populations may experience limited diagnostic capacity, both prenatal and postnatal, and limited access to or uptake of expert care services leading to high mortality (Higashi et al., 2015; Sitkin, Ozgediz, Donkor, & Farmer, 2015). In addition, affected children in LMICs may have limited support systems and experience stigmatization, isolation, and abandonment (Claude, Juvenal, & Hawkes, 2012; Sitkin et al., 2015; WHO, 2011).

Despite this high burden of CAs, many LMICs do not have rigorous CA surveillance programs that can provide accurate data to inform prevention and implement care measures (Ajao & Adeoye, 2019; Zaganjor et al., 2016). Prioritization of surveillance programs may increase as countries measure their progress toward meeting Sustainable Development Goal targets (UN IGME, 2018). The WHO Birth Defects Resolution WHA63.17 from the 2010 World Health Assembly called for the strengthening of birth defect surveillance systems globally and nationally (WHO, 2010). The recent Zika virus outbreak in the Americas led to many babies being born with CAs of the brain and eyes and neurodevelopmental sequelae—congenital Zika syndrome. The Zika epidemic and the 2018 safety alert from Botswana of the possible increased prevalence of neural tube defects associated with periconceptional use of dolutegravir (an HIV treatment drug) have further emphasized the importance of strengthening surveillance systems in LMICs (Larrandaburu et al., 2018; Zash et al., 2019). Currently, the COVID-19 pandemic is creating a further reason to study the impact on the risk of CAs from the infection itself, associated fever (Moretti, Bar-Oz, Fried, & Koren, 2005), or the treatments used (Louchet et al., 2020), and from a change in other risk factors due to the healthcare and socioeconomic consequences of the pandemic.

One problem that hampers CA surveillance in LMICs is the difficulty of collecting accurate data on CAs in the population. Identifying and describing CAs, and coding and classifying CAs, typically require a level of training and knowledge that may be challenging to develop and sustain in low-resource settings where access to specialists is limited. Currently, two main approaches are being used to tackle this problem. The first involves the health care staff at the point of care (e.g., birthing center) taking and sending photos to expert clinicians (e.g., medical geneticist or pediatrician) at a central level who make a final diagnosis. The second involves the staff at the point of care making use of a CA atlas (a set of pictures and drawings of CA, their names, and the disease codification) to facilitate the description and coding of CA, either the WHO atlas available in paper or electronic pdf form (WHO, 2014) or the ECLAMC electronic database (ECLAMC, 2013). Some surveillance systems have also developed their own atlases (Global Birth Defects, 2020).

The proliferation of mobile phones in LMICs in recent years has provided better opportunities to improve healthcare delivery and health surveillance in these settings using mobile health (mHealth) (Brinkel, Krämer, Krumkamp, May, & Fobil, 2014; Wallis, Blessing, Dalwai, & Do Shin, 2017). In this paper, we describe the global birth defects (GBD) application, an innovative mobile application specifically designed for use in surveillance/research programs operating in low resource settings to facilitate their accurate description and coding of externally visible CAs at birth. The app was developed as part of a coordinated response to the recent Zika virus outbreak in Latin America.

## 2 | METHODS

### 2.1 | Project team

The project team consisted of an international expert committee, research team, software development experts, and foreign language translators.

At the start of the project, the International Committee on Congenital Anomaly Surveillance Tools (ICAST) was formed [members in authorship]. This expert committee was designed to cover most of the world regions (Latin America, Africa, Asia, Europe, and United States), most of the international congenital anomaly surveillance networks (ICBDSR, EUROCAT, WHO South East Asia surveillance systems, ECLAMC), and disciplines necessary for the design of the app (particularly in clinical genetics, user experience in low resource settings, and public health surveillance). The purpose of the committee was to contribute wide expertise and experience to the development of the tool, so that it could be launched as a credible global tool for CA surveillance. The committee met mainly by teleconference and twice face-to-face. At the second face-to-face meeting, the committee provided an early expert testing review of the app prototype.

### 2.2 | Selection of congenital anomalies and information content

Lists of major external congenital anomalies for surveillance were compiled from the WHO Atlas (WHO, 2014), EUROCAT subgroups (EUROCAT, 2013), and the clinical literature, and submitted to the International Committee for comment in order to derive the final list for inclusion (see Table 1). Criteria for inclusion, derived from committee discussions, were as follows:

- Clinical and public health impact: major congenital anomaly
- Modifiable: amenable to primary prevention and care
- Ease of diagnosis: externally visible, identifiable at birth
- Frequency: relatively common (likely to be seen annually in very large hospitals)
- Codable: associated with an international classification of disease (ICD) v10 code.

These criteria did not all have to apply for a specific CA to be included. For example, some anomalies were rare subtypes of larger categories, but were included for completeness and to avoid misclassification (e.g., iniencephaly), a rare form of neural tube defect, and some rare types of encephalocele.

Some common minor anomalies were included in order to help the user arrive at an “answer” to their question of identifying the congenital anomaly seen, also giving an opportunity to instruct the user not to include the anomaly (if isolated) in surveillance data and preventing misclassification as major anomalies. Minor anomalies are defined as those that do not have significant surgical, medical, or cosmetic importance. Minor anomalies were selected from the lists of minor anomalies compiled by EUROCAT (EUROCAT, 2013) and the WHO Atlas (WHO, 2014).

Syndromes were restricted to common syndromes that are readily identifiable at birth, both genetic syndromes and congenital infection syndromes. Because of its public health importance, fetal alcohol spectrum disorder is also included, although diagnosis at birth is difficult. Because the impetus to develop the app was the Zika virus epidemic, special attention was paid to defects associated with Zika virus infection. The syndromes resulting from congenital infections (e.g., congenital syphilis, toxoplasmosis, and cytomegalovirus) were included because of their public health importance, even though some are difficult to identify at birth.

Diagrams and photos of the anomalies were sourced mainly from the WHO and ECLAMC atlases (ECLAMC, 2013; WHO, 2014). Other sources included open access medical journal papers, reference books, and the CDC-Beijing Medical University collaborative project photo library. When available, we used diagrams instead of photos and only used both when they each depicted different relevant identification features or aided recognition of the anomaly.

Each anomaly was coded using the International Classification of Disease v10 (ICD10) code with British Pediatric Association (Royal College of Pediatrics and Child Health, RCPCH) one-digit extension, along with a simple description to ease the understanding for nonexperts. ICD10 codes for the few minor anomalies included in the app were omitted to emphasize that they are not to be reported to surveillance systems when found in isolation.

To complement the images and descriptions, we also identified and included relevant neonatal examination videos and links to further resources.

### 2.3 | Recording data: "Basic" versus "surveillance" versions

Our original idea was to develop one version of the app that can be used for both surveillance/research and training. However, use of the app for surveillance requires data collection and a strict mechanism for data protection, which impedes its more general availability for training. We therefore created two versions of the app, to which access is determined by the registration code used:

- The *Basic version*, which is designed for use by persons with an interest in understanding/improving congenital anomaly diagnosis or coding, including for training purposes, or for surveillance programs that do not need the data collection functionality.
- The *Surveillance version*, which is an extension of the Basic version that allows recording of pseudonymous data for each baby for use in surveillance/research programs. These data can be uploaded to a secure server in a section to which only the surveillance program itself has access and can be downloaded (as Excel files) by the surveillance program.

### 2.4 | Ethics and data protection aspects

Ethical approval for the development of the app was obtained from Ulster University Research Ethics Committee.

The first ethics issue was use of images in the app pages. Most of the images used in the app were already available in the public domain. However, permission was obtained for all images included in the app from the source (WHO, ECLAMC, CDC, journal authors) who in turn had appropriate permission from parents. When possible, we concealed the identity of the baby by pixilating parts of the image (particularly facial features) or showing only an image of the relevant body region. We also excluded particularly distressing or undignified images. As mentioned, when appropriate, we utilized diagrams over photos. All sources of images were fully referenced in the app.

The second ethics issue concerned recording data with the “Surveillance” version of the app. All surveillance programs interested in using the app are required to first obtain local ethics or institutional review board or nationally required permission. Only pseudonymized data can be collected with the app (i.e., using a study unique identification code for the individual), rather than any personal identification details. The test version of the “Surveillance” version of the app also enables photos to be taken of the baby for validation purposes during field-testing. Those requiring access to the photo-taking function of the app must have this covered in their ethical clearance, as well as a procedure for obtaining informed consent from parents. Users are also required to verify that consent has been obtained (a tick box) at the time a photo is taken.

We included a registration process that uses a registration code. Access to the specific version of the app is determined by the registration code used. To prevent casual use of the app, the registration code for the Basic version (one code: XJNL) is made available only at the GBD website (https://globalbirthdefects.tghn.org/download-birth-defects-surveillance-app/launch-basic-version-app/), not in the app store. Institutions with permission to use the Surveillance version of the app receive a unique registration code for their institution, which unlocks data recording functions. The institutional user regulates access to the app by the distribution of its unique code to its own staff. A limited amount of data collected as part of the registration process is meant to establish users' statistics and provide support when needed. All users must read and agree to the data protection statement (https:/globalbirthdefects.tghn.org/download-birth-defects-surveillance-app/app-privacy/policy/) before completing the registration process.

During use, a pin code chosen by users provides timed access to the app, to prevent the data entered being visible to others who might use the device afterwards.

### 2.5 | Design and user experience

The app was designed for use in both Android and Apple (IOS) devices. Bearing in mind the limitation of internet access in low resource settings, the app was designed to be operated offline. However, the user will need internet for downloading the app or uploading data into a server (for those using the “Surveillance” version). The app allows multiple user accounts on the same device, particularly for use in tablets where hospitals may provide one tablet for multiple users.

The anomalies are organized according to the region of the body affected because the app is designed to be used for identification of what is observed neonatally when examining the baby, not for later classification by pathogenesis or etiology. On the app home page, we use an image of a baby (which can flip between front and back) from where users can easily locate the region of the body affected, see the various options, and navigate through to the specific CA. We also include a mechanism that allows users to distinguish between similar anomalies, a simplified “differential diagnosis,” by moving between them easily. In the “Surveillance” version, the user can choose to record the defect, and the app automatically records the ICD10 code. Multiple anomalies can be recorded per baby.

We translated the app into Spanish and Portuguese with future additional translations envisaged, with the language being automatically chosen depending on the phone settings.

### 2.6 | Software development

The app was written in C# with Xamarin.ios and Xamarin.android using Visual Studio for MAC IDE (all Microsoft).

**FIGURE 1:**
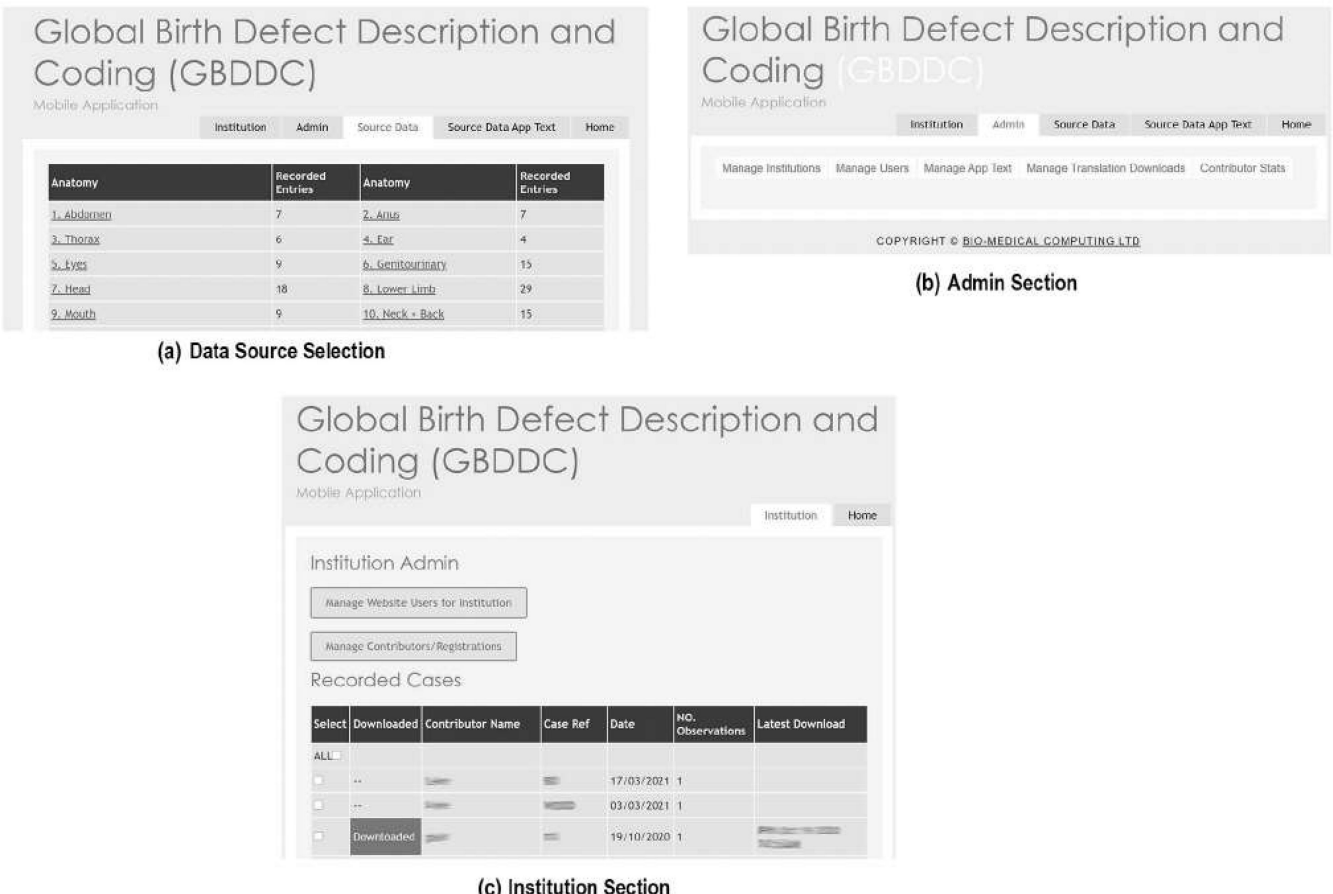
(a-c) Sections of the GBDDC app software management website

A website was created to host and manage all operations of the app. The website has three main sections (Figure 1):

1. The “source data” section hosts a database of the main app contents, which can be updated by the research team (adding or deleting photos, adding or deleting text), the contents of the database fields and images are exported into the development workflow and incorporated into new versions of the App to reduce transcription error.
2. The “admin” section allows the app administrator to download usage statistics, manage the app texts (including importing translated texts), and manage institutions (surveillance/research programs) and their designated users who have been assigned access to the Surveillance version of the app.
3. The “institution” section acts as secure online data platform hosting data for institutions using the "Surveillance” version of the app—each institution has a unique section within the platform and data are not visible to the International Committee for Congenital Anomaly Surveillance Tools (ICAST) team. The secure server is located in the UK. The surveillance program data manager can access the site to see the data files which have recently been uploaded (each case is a separate file in Excel), and download and delete the files. After downloading, the files can be matched to other data held on the baby according to the unique reference number of the baby. The surveillance management website is written in ASP.NET (C#, html, javascript) using the same SQL Server Database as the web service used to collect data online.

There are two options for the “institution” section of the server. The surveillance program can use its designated section of the central server to upload and download data. Alternatively, the app can be customized to point to an alternative server, and the “admin” and “institution” website can be copied to that server. In terms of sustainability, the central server option requires a small yearly fee to rent the server covering all users, while the customization for a different server requires some initial IT support. Changes to the app contents (photos, text, etc.) continue to require ICAST approval for quality control.

### 2.7 | Expert review and field testing

Once development of the Basic version of the app was completed by the project team, it was made available for expert review. We sent invitations, along with an electronic review questionnaire and the registration code, to an established list of birth defects experts beyond the International Committee. The expert reviewer list constituted by the International Committee included experts from the ECLAMC network, the WHO Technical Working group on birth defects, and other birth defect surveillance systems in LMICs and developed countries. A total of 26 experts were invited, and 12 (46.2%) participated in the review. The 12 reviewers came from Africa, Europe, North America, South America, and Australia. Using the questionnaire, which included both closed questions and space for comments, the reviewers provided feedback on the general design and functionality of the app as well as its contents. After receiving feedback from 12 reviewers (see Report at https://globalbirthdefects.tghn.org/download-birth-defects-surveillance-app/), the app was revised to create a final version.

Once the “Basic” version was ready, we finalized development of the surveillance components. The complete “Surveillance” version of the app was then made available for testing by selected congenital anomaly surveillance programs in Africa and South America. The testing both allows validation of the birth defect “diagnoses" made by users by comparing these with photos taken of the babies during field testing and reviews the operational aspects of the app regarding uploading and downloading data and other processes. Field-testing is currently ongoing.

### 2.8 | Dissemination and training

The app has been available for download from the Google Play Store and Apple App Store since December 2019.

While “congenital anomaly” is now the preferred term used by the WHO and many surveillance networks, we used the term “birth defects” in the name of the app and associated resources because of its greater recognition in the wider public health field. Birth defects, although often used interchangeably, particularly in the United States, is a wider term which sometimes includes other conditions, such as metabolic defects, which are not included in the app.

We created the GBD site on the Global Health Network to include both an extensive inventory of birth defects surveillance tools, and to facilitate dissemination and provide training in use of the app. The app section of the website includes the “Basic” version registration code, how to apply for and access the “Surveillance” version, and a series of instructional videos (https://globalbirthdefects.tghn.org/download-birth-defects-surveillance-app/launch-basic-version-app/) on the following topics:

- Introducing the basic version of the GBDDC app https://youtu.be/wzZ5-Ambc-g
- Innovative solutions: introducing our app https://youtu.be/Mdf7F8i7azg
- How to download and register for the apphttps://youtu.be/HcayOg7Go3s
- How to navigate, record and upload data https://youtu.be/d4LM5BLpxPQ
- Recording data for congenital Zika syndrome and microcephaly https://youtu.be/VI-d61SeViE

We targeted and made presentations about the app to experts on CA, students, and other relevant stakeholders from LMICs during annual professional meetings and conferences, including ZikaPLAN meetings; ICBDSR conferences and training workshops on Birth Defect Surveillance and Prevention; Latin American ECLAMC/RELAMC meetings; and the International Conference on Birth Defects and Disabilities in the Developing World in Colombo, Sri Lanka, in 2020. We also held webinars to present the app on World Birth Defects Day (3 March) in collaboration with other stakeholders. Our International Committee and Expert Reviewers also helped in dissemination of the app within their networks. An African network of Congenital Anomaly Surveillance, Prevention and Care is starting in 2021, which will help disseminate the tool in Africa.

## 3 | RESULTS

**FIGURE 2:**
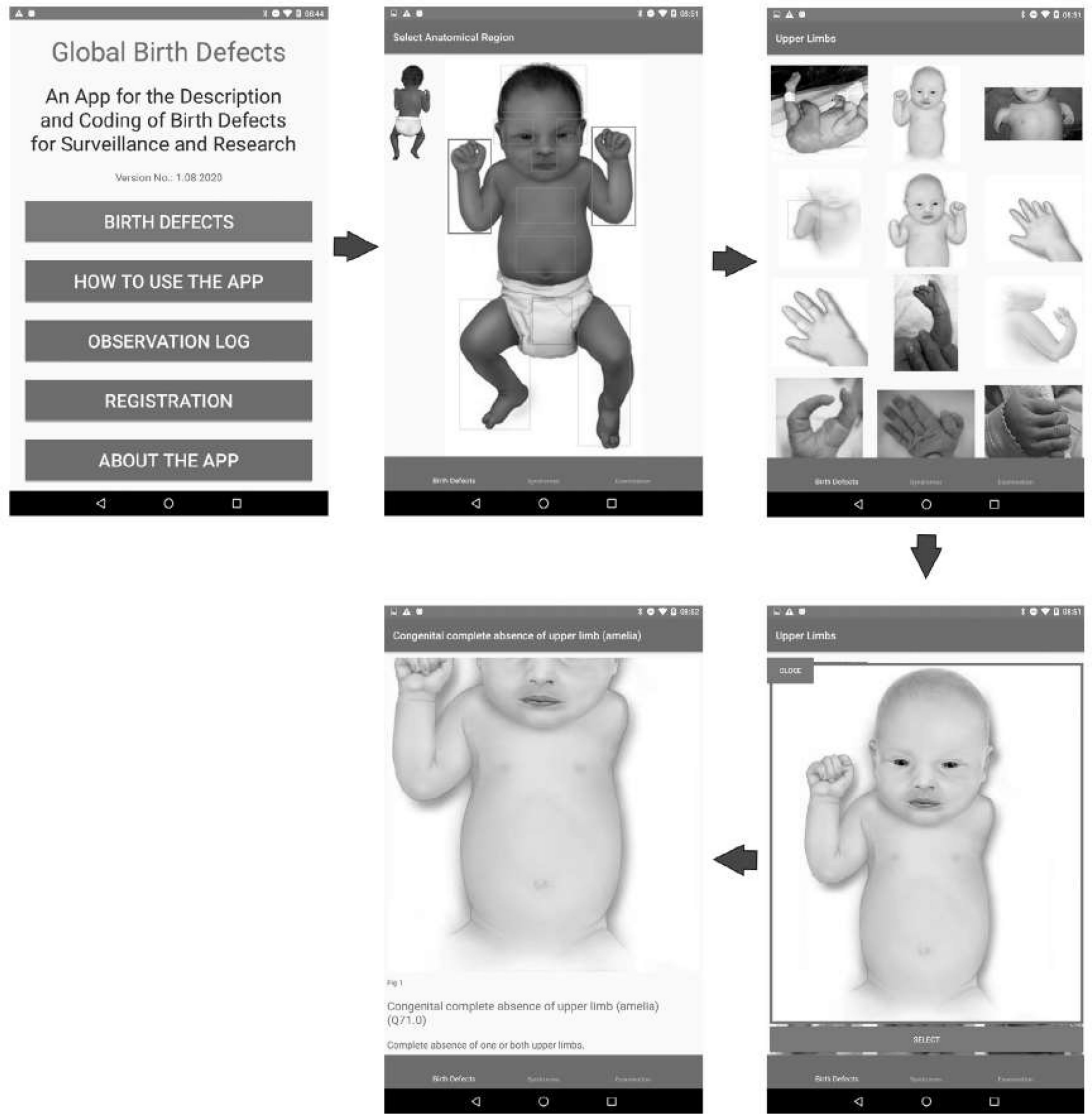
Birth defect selection pathway (e.g., Congenital complete absence of upper limb—Q71.0)

**FIGURE 3:**
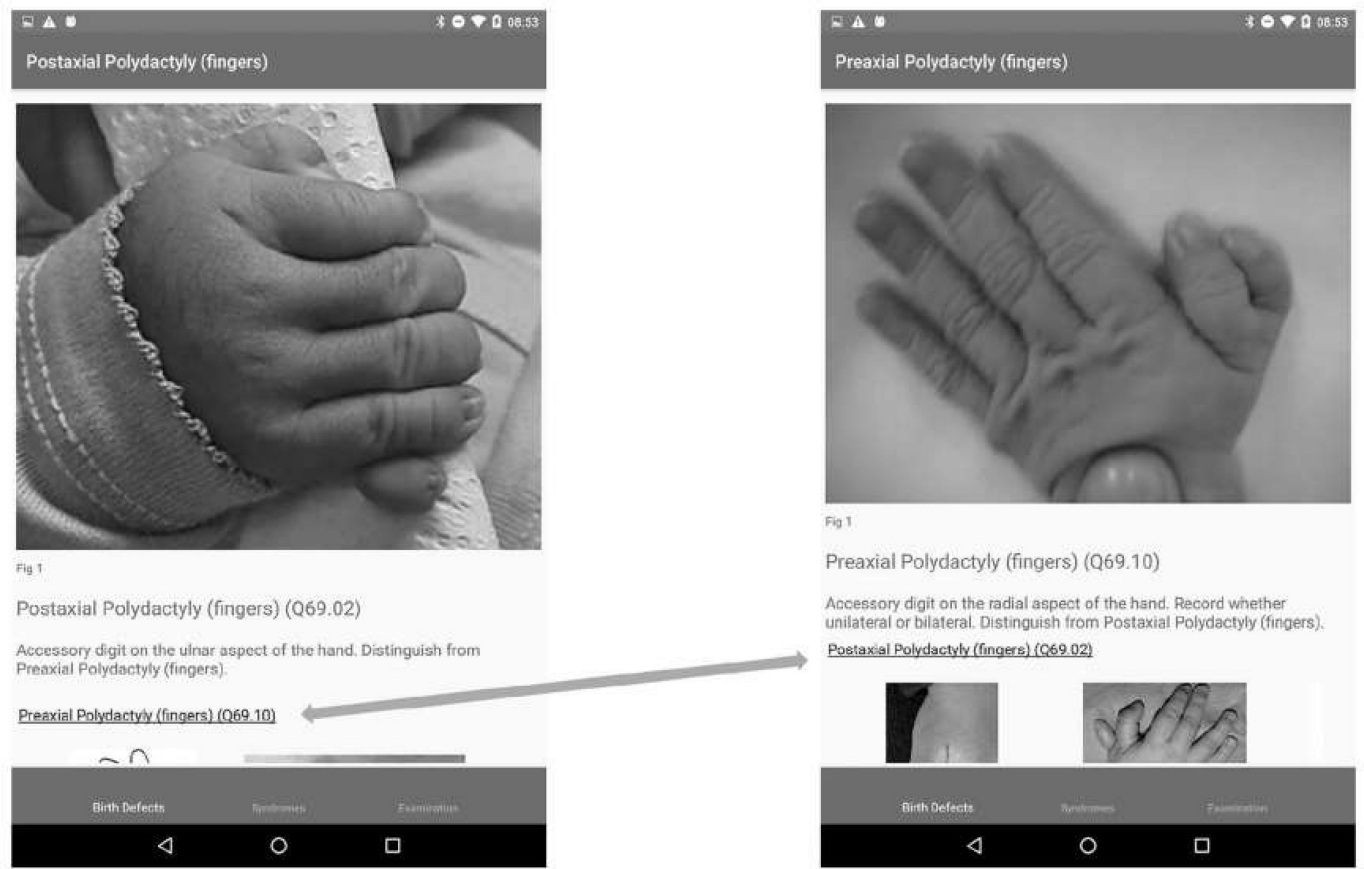
“Differential diagnosis” of postaxial and preaxial polydactyly of fingers

A total of 98 (88 major and 10 minor) externally visible anomalies and 12 syndromes or rare conditions are included in the app (see Table 1). Information on each specific anomaly includes the anomaly term, the ICD10-RCPCH code (concealed for minor anomalies), a brief description and links to further resources when available, images (including subcategories when available), and sources of the images. Figure 2 shows an example of the navigation pathway to a single anomaly. Figure 3 shows a “differential diagnosis” example.

One suitable neonatal examination video from the WHO was identified (WHO/TDR, 2012) and included in a separate tab, cut into subsections including preparations prior to the examination, examination of different body regions with guidance on possible abnormalities, and post examination procedures.

The syndromes section of the app (accessible via a separate tab) emphasizes the challenge in diagnosing syndromes and provides users with links to additional resources. This section includes chromosomal syndromes (e.g., Down syndrome, Trisomy 13 and 18), skeletal dysplasias, and congenital infection syndromes. The most common component anomalies are listed with a link for each to the main part of the app. In the “Surveillance” version, the user is encouraged to record each component anomaly separately, as well as the syndrome diagnosis. Skeletal dysplasias are included as a single condition with the overall ICD code because the Committee (ICAST) concluded that it is too difficult for nonexperts to differentiate well between skeletal dysplasia types and differentiation often is dependent on imaging, but photos of the different types are included. The syndrome section also includes some rare but easily identifiable conditions that involve multiple systems that are not syndromes—conjoined twins and acephalus acardia.

**FIGURE 4:**
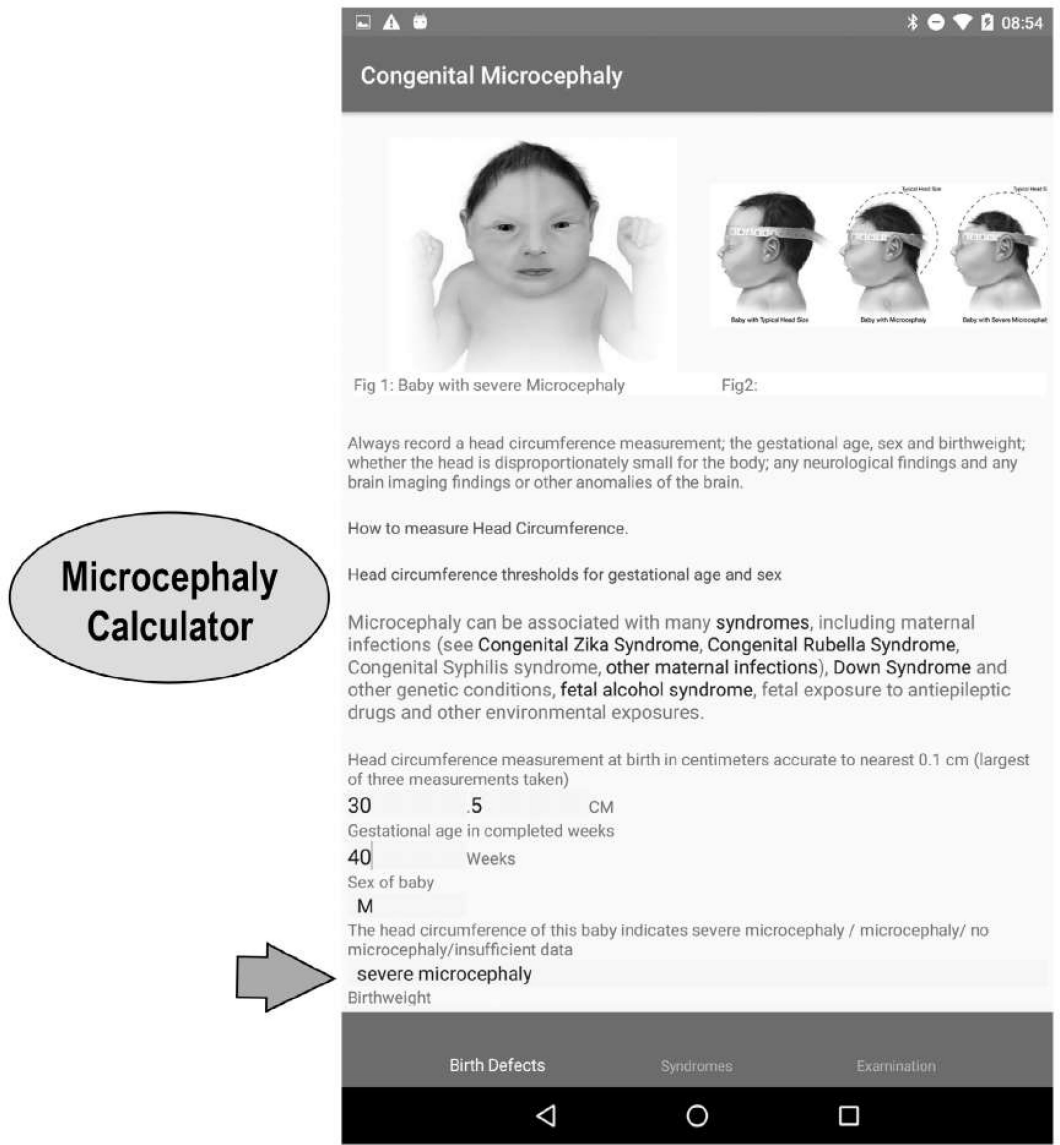
Microcephaly page showing severity calculator (Data entered indicate baby has severe microcephaly)

Particular attention is given to congenital Zika syndrome (CZS) and microcephaly because the app was in response to the Zika virus epidemic. We emphasize the fact that the diagnosis of CZS is based on both the presence of maternal Zika virus infection during pregnancy (maternal report and/or laboratory confirmation) and presence of microcephaly or other neural or ocular anomalies. Links to guidance materials from the WHO and CDC are provided. The “surveillance” version requests that information on both infection and anomalies present is recorded and allows the surveillance program to decide whether cases meet the CZS criteria. The microcephaly page has videos on head circumference measurement and Intergrowth and WHO charts for head circumference thresholds. A “microcephaly calculator” automatically classifies babies into normal head circumference (>−2SD), microcephaly (−3SD to −2SD) and severe microcephaly (<−3SD) based on information entered on the head circumference (in cm with one decimal point), gestational age, and sex of the baby (Figure 4). This calculator is available in both the “Basic” and “Surveillance” versions.

The majority of the expert reviewers of the “Basic” version of the app felt it was easy to download (58.5%) and easy to register (66.5%) to use it. Android users found it challenging accessing the app from the app development website, but this is now overcome as it is in the app stores. Some reviewers observed that the app took a long time to download even with use of Wi-Fi, and the app slowed their phones down by taking over the phone memory. Further software development was carried out to reduce the likelihood of a crash in devices with 1 GB of RAM or less, but because of the need to view high resolution images offline, there was a limit to the extent to which the demand on memorv could be reduced.

Hundred percent of reviewers felt the clinical content of the app was very appropriate (41.7%) or appropriate (58.3%) for nonexpert users. On average, 66.0% of the reviewers rated the contents of each body part as needing minimal improvement. The reviewers suggested improvements to the current contents and proposed several conditions to be added to the app. These conditions were reviewed by the Committee for potential inclusion, and three were included. A full report of the expert review is available (https://media.tghn.org/medialibrary/2020/11/Expert_Review_of_Basic_Version_of_App.pdf). Review by the intended users is part of the Phase 2 review of the Surveillance Version of the App.

**FIGURE 5:**
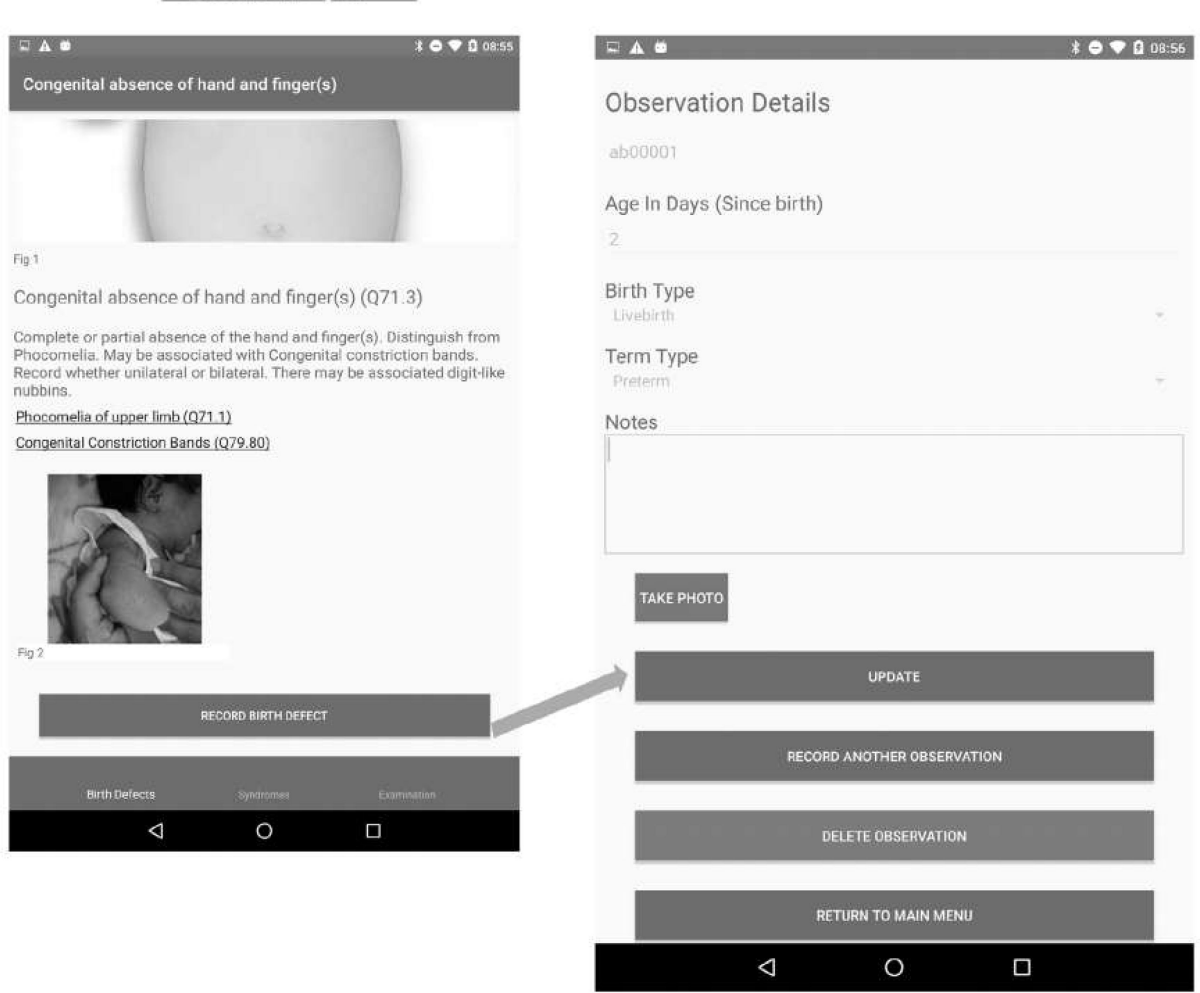
Surveillance version data-recording page

The data set of the “Surveillance” version includes ICD10-RCPCH code (automatic), congenital anomaly term (automatic), date and time of recording (automatic), pseudonymized reference number of the baby (or barcode reader), age in days when diagnosed (completed days since birth), whether live-born or stillborn, whether term/post-term or preterm, and free text to describe the anomaly in more detail (Figure 5). Multiple anomalies can be recorded per baby. Each body region also has an “Other” category in which information about other anomalies can be recorded. For microcephaly, the data entered also include sex of the baby, gestational age, and head circumference, with these data being deposited in the text field of the data set. For CZS, the data set includes evidence for Zika virus infection, in separate free text fields for laboratory evidence and non-laboratory evidence.

## 4 | DISCUSSION

### 4.1 | The app in the context of mHealth in LMIC countries

mHealth interventions have become increasingly popular in LMICs (PricewaterhouseCoopers, 2012; Wallis et al., 2017), providing opportunities for healthcare delivery as well as surveillance and research. Of the over 165,500 existing mHealth interventions in 2015 with unrestricted access, 7% were focused on matemal/neonatal health problems, such as educating women, improving service utilization and adherence to treatment plan (Chen, Chai, Dong, Niu, & Zhang, 2018; Murray & Jennifer, 2015; Sondaal et al., 2016). A 2014 review found 9 mHealth interventions focused on public health surveillance in Sub-Saharan Africa (Brinkel et al., 2014). These were mainly for infectious disease surveillance, but also child malnutrition and maternal health, with the majority being in a pilot phase. WHO South East Asia has developed an app for CA surveillance for use by WHO SEARO participating hospitals (WHO South-East Asia, 2020) focusing on data capture rather than providing pictorial aids. The GBD app is the first globally available app to focus on CA diagnostic aspects within a surveillance context.

Most mHealth interventions in LMICs end at the pilot stage and are rarely brought to scale (GSMA, 2020; Wallis et al., 2017). Regulatory challenges include lack of appropriate data privacy and protection legislation (Cory & Stevens, 2020) and problems with sharing patient data using mHealth technology (Wallis et al., 2017), crossborder interoperability or standards, risks associated with technology, such as theft, malware and device sharing, and difficulty in integrating mHealth into existing health systems (Wallis et al., 2017). Technological challenges include availability of mobile cell phone signal, broadband signal coverage, and cost of data, electricity, and interoperability with existing systems (GSMA, 2020; Wallis et al., 2017). User factors that may influence uptake of apps include issues, such as username/password and simplicity (user-friendliness) of the technology, difficulty in changing behavior and limited ongoing site support (Wallis et al., 2017).

In the development of the GBD app, we took into consideration these major challenges. We introduced flexible data protection mechanisms (e.g., creation of two versions of the app—one requiring no data collection and hence easy access to the registration code and another with data collection functionality and a more restrictive access to the registration code); reduced the internet access barrier by allowing offline use of the app; simplified the design to facilitate use by nonexperts (e.g., pictorial navigation, easy to understand description of the anomalies, and automatic capture of ICD10 code); devised an extensive dissemination plan targeting relevant stakeholders; and provided training and support to users via instructional videos. However, some mHealth challenges (e.g., lack of a national data privacy and protection policy) can only be handled by national governments or through wider global efforts. A challenge exists in designing an app for surveillance purposes where the user may need it very infrequently, or staff turnover is high, requiring a simple interface and minimal training.

The "Basic" version of the app has, as of October 2020, been downloaded in 39 countries, without reports of problems with the technology. A survey of users is intended. The “surveillance” version of the app is currently undergoing field-testing to assess the suitability for the target users of both the clinical identification aspects, and the data recording aspects for both user and data manager. One of the major challenges in its design is providing interoperability with existing surveillance programs. We considered several options. One of the options was to agree to a core surveillance data set regarding mother and baby. We considered this unfeasible, however, due to the different objectives of surveillance and research programs worldwide that necessitate different data items, the greater data protection problems associated with a full surveillance data set, and the fact that often surveillance and research requires data collection on babies without CAs as well as those with CAs (e.g., data on exposure of the mother to medication). We chose to record only pseudonymized data associated with the CA diagnosis itself, not risk factors. These diagnostic data can be downloaded from the central server by the surveillance program and matched with all other data collected on each CA case. This requires some informatics expertise for the surveillance program to operate the downloading and matching of files. It also requires the surveillance program to operate an accurate system for pseudonymizing via a reference number or bar code (e.g., bar codes used by the Ugandan surveillance program) (Mumpe-Mwanja et al., 2019). A further option is a more bespoke program-by-program integration with existing systems. We are exploring these options in the field during 2021, having been delayed by the Covid pandemic, and plan a report of field testing by the end of 2021.

### 4.2 | Strengths, limitations, and future directions

One of the most difficult areas of the app is the distinction of its clinical and surveillance/research use. We have been careful to talk about “Description and Coding” rather than “Diagnosis” where possible, to avoid it becoming a tool for the overconfident, nonexpert diagnosis of congenital anomalies. On the other hand, the app can be used as part of health professional training, and when complemented with other resources, facilitate diagnostic training.

Recognizing the complex nature of syndromes and expertise including laboratory results/genetic testing needed for their diagnosis, we included in the app only easily recognizable syndromes. Further validation testing may allow us to see how well nonexperts can classify babies to these syndromes. Importantly, the app encourages all syndrome features (component anomalies) to be recorded, not just the overall diagnosis.

Minor anomalies have clinical value in helping to identify a syndrome, or the potential for delayed neurodevelopment or sensorineural anomalies, or a higher risk of nonvisible internal major anomalies. However, their clinical value must be distinguished from their value in epidemiologic surveillance. The potential for great diagnostic variability in clinical practice (outside specific research situations) and the aim to focus resources on conditions of medical importance, combined with the large numbers of babies with minor anomalies (about 15–20% of all infants (EUROCAT, 2013) have led most surveillance programs to recommend the exclusion of isolated minor anomalies, based on exclusion lists (EUROCAT, 2013; WHO 2020c). The argument for including common minor anomalies in the GBD app was that there was training value in being able to identify a common minor anomaly seen at birth, and be instructed that it is an anomaly for exclusion from surveillance, rather than risking the app user opting for the major anomaly of “closest fit.” We had feedback from one surveillance program in Africa that suggested that over-recording of minor anomalies was a problem that this app could help with. To avoid inadvertently encouraging the recording of isolated minor anomalies, however, no ICD code is given. On the other hand, the app should foster the development of good observational skills and description to help with referral to experts, and minor anomalies can be recorded in free text. The app should encourage the user who observes multiple minor anomalies to conduct a more intensive investigation of the baby. A section of the “syndrome” tab is under development to help with dysmorphological description.

Lacking in the GBD app are resources that help researchers and health professionals talk to parents about their baby's condition. While referral to experts is needed for discussion with parents of issues of treatment, possible causes, and recurrence risk, there may nevertheless be a case for helping nurses and midwives start a sensitive discussion and correct any false preconceptions about the causes of a CA (Bello, Acquah, Quartey, & Hughton, 2013; Claude et al., 2012).

Within the vision of Triple surveillance (surveillance, prevention and care) (Botto & Mastroiacovo, 2018), this app, or a set of interoperable apps, could potentially be expanded beyond CA surveillance to include prevention and care. While our app is designed for nonexpert use in surveillance/research situations, or as a basic training tool, a need for more sophisticated tools exists that can help clinicians in their practice regarding diagnostic assessment and treatment options. Such expert assistance tools, which also enter the domain of artificial intelligence (AI), are in rapid development across many areas of medicine. One such is the Face2Gene App (Gurovich et al., 2019), which, by photographing the face of the patient, and recording other observations, offers a range of possible genetic syndrome diagnoses. Developing the knowledge of health professionals regarding birth defects prevention is highly encouraged. Currently, the GBD app signposts users to the accompanying resources inventory on the GBD website (https://globalbirthdefects.tghn.org/resources-inventory/) regarding existing tools for congenital anomaly prevention.

The app is only helpful to the extent that it improves the accuracy and completeness of case finding. Current field-testing initiatives will help to establish this and guide improvements. The app encourages users to enter detail in free text, and further development where appropriate consent processes are implemented for photo taking, is under consideration. Taking photos with the app would improve data protection compared with current practices.

The GBD app only includes externally visible major anomalies as it is designed for use by nonexpert health professionals or nurse researchers, who will see the baby at birth and record what they see, but not have immediate access to further diagnostics, such as ultrasound, X-Ray, echocardiograms, and so forth. Nevertheless, as access to these technologies widens, further development to incorporate simple diagnostics is possible, and indeed the revised WHO Atlas (now called the Quick Reference Handbook, part of the Birth Defect Surveillance Manual WHO, 2020c) also includes the most common heart anomalies. Plans are ongoing to further develop the GBD app as a companion tool to the WHO Quick Reference Handbook.

## 5 | CONCLUSION

The global development of CA surveillance is highly encouraged in order to support prevention and care and thus improve child health outcomes. The GBD app is an mHealth tool to facilitate the description and coding of externally visible congenital anomalies at birth for surveillance and research in low resource settings and training of health professionals. While designed in response to the Zika virus epidemic, it has broader application to CA surveillance. A “Basic” version has been released, while a “Surveillance” version that allows data collection is undergoing field-testing. Further development of the app to support prevention and care, and respond to emerging health threats, is possible.

## Figures and Tables

**TABLE 1:** List of anomalies and syndromes included in the global birth defects app

Body region	Name of anomaly	ICD10-RCPCH code
**Head**	Anencephaly	Q00.0
	Craniorachischisis	Q00.1
	Iniencephaly	Q00.2
	Frontal encephalocele	Q01.0
	Nasofrontal encephalocele	Q01.1
	Occipital encephalocele	Q01.2
	Parietal encephalocele	Q01.80
	Orbital encephalocele	Q01.81
	Nasal encephalocele	Q01.82
	Aplasia cutis congenita	Q84.80
	Congenital microcephaly	Q02
	Congenital hydrocephalus	Q03.9
	Holoprosencephaly	Q04.2
	Abnormal head shape at birth^a^	
	Craniosynostosis	Q75.0
	Other head	
**Neck and Back**	Cervical spinal bifida with hydrocephalus	Q05.0
	Cervical spinal bifida without hydrocephalus	Q05.5
	Thoracic spinal bifida with hydrocephalus	Q05.1
	Thoracic spinal bifida without hydrocephalus	Q05.6
	Lumbar spinal bifida with hydrocephalus	Q05.2
	Lumbar spinal bifida without hydrocephalus	Q05.7
	Sacral spinal bifida with hydrocephalus	Q05.3
	Sacral spinal bifida without hydrocephalus	Q05.8
	Caudal regression	Q76.41
	Other neck and back	
**Mouth and nose**	Cleft palate	Q35
	Unilateral cleft lip	Q36.9
	Bilateral cleft lip	Q36.0
	Median cleft lip with or without cleft palate	Q36.1
	Cleft palate with bilateral deft lip	Q37.8
	Cleft palate with unilateral cleft lip	Q37.9
	Pierre Robin sequence	Q87.08
	Hemifacial microsomia	Q67.4
	Agenesis/underdevelopment of nose	Q30.1
	Fissured, notched and cleft nose	Q30.2
	Other mouth and nose	
**Eyes and ears**	Anophthalmia	Q11.1
	Microphthalmia	Q11.2
	Congenital cataract	Q12.0
	Coloboma of iris	Q13.0
	Cryptophthalmos	Q11.2
	Eyelid malformation^a^	
	Aniridia	Q13.1
	Epibulbar dermoids	D31.9
	Anotia	Q16.0
	Microtia	Q17.2
	Other eyes and ears	
**Chest**	Ectopia cordis	Q24.8
	Poland sequence	Q79.82
	Accessory nipple^a^	
	Other chest	
**Abdomen**	Gastroschisis	Q79.3
	Omphalocele	Q79.2
	Prune belly sequence	Q79.4
	Limb body wall	Q79.5
	Umbilical hernia^a^	
	Other abdomen	
**Anal**	Anal atresia with fistula	Q42.2
	Anal atresia without fistula	Q42.3
	Other anal	
**Genitourinary**	Cloacal exstrophy	Q64.10
	Bladder exstrophy	Q64.1
	Hypospadias	Q54.0, Q54.1, Q54.2
	Indeterminate sex	Q56.4
	Undescended testicle unilateral^a^	
	Undescended testicle bilateral^a^	
	Hydrocele^a^	
	Inguinal hernia^a^	
	Other genitourinary	
**Lower limb**	Arthrogryposis	Q74.3
	Talipes equinovarus	Q66.0
	Pterygium of joints	Q74.8
	Syndactyly fused toes	Q70.2
	Syndactyly webbed toes	Q70.3
	Synpolydactyly/polysyndactyly (toes)	Q70.4
	Syndactyly of second and third toes^a^	
	Split foot	Q72.7
	Preaxial polydactyly (toes)	Q69.20
	Postaxial polydactyly (toes)	Q69.22
	Congenital constriction bands	Q79.80
	Sirenomelia	Q87.24
	Congenital dislocation of knee	Q68.20
	Congenital overgrowth of limbs	Q74.81
	Congenital dislocation of hip (unilateral)	Q65.0
	Congenital dislocation of hip (bilateral)	Q65.1
	Congenital complete absence of lower limb	Q72.0
	Phocomelia of lower limb	Q72.1
	Congenital absence of both lower leg and foot	Q72.2
	Congenital absence of foot and toe(s)	Q72.3
	Congenital absence or hypoplasia of toe(s) with remainder of foot intact	Q72.30
	Absence or hypoplasia of first (great) toe with other digits present	Q72.31
	Longitudinal reduction defect of femur	Q72.4
	Longitudinal reduction defect of tibia	Q72.5
	Macrodactyly (toes)	Q74.04
	Other lower limb	
**Upper limb**	Arthrogryposis	Q74.3
	Congenital complete absence of upper limb	Q71.0
	Phocomelia of upper limb	Q71.1
	Congenital absence of both forearm and hand	Q71.2
	Congenital absence of hand and finger(s)	Q71.3
	Congenital absence of fingers	Q71.30
	Absence or hypoplasia of thumb	Q71.31
	Longitudinal reduction defect of radius	Q71.4
	Split hand	Q71.6
	Preaxial polydactyly (fingers)	Q69.1
	Postaxial polydactyly (fingers)	Q69.0
	Syndactyly fused fingers (with synostosis)	Q70.0
	Syndactyly, webbed fingers (without synostosis)	Q70.1
	Synpolydactyly/polysyndactyly (fingers)	Q70.4
	Macrodactyly (fingers)	Q74.04
	Pterygium of joints	Q74.8
	Congenital constriction bands	Q79.80
	Other upper limb	
**Syndromes/rare**	Trisomy 21 (Down syndrome)	Q90
	Trisomy 18 (Edwards syndrome)	Q91.3
	Trisomy 13 (Patau syndrome)	Q91.7
	Congenital Zika syndrome	P35.4
	Congenital Zika syndrome—suspected maternal Zika virus infection	Z20.8
	Congenital Zika syndrome—laboratory confirmed maternal Zika virus infection	U06.9
	Congenital rubella syndrome	P35.0
	Skeletal dysplasias	Q77
	Other congenital infections (congenital cytomegalovirus, congenital toxoplasmosis, congenital syphilis)	P35.1, P37.1, A50.9
	Conjoined twins	Q89.4
	Acephalus acardia	P02.3
	Fetal alcohol syndrome or spectrum disorder	Q86.0
	Congenital skin disorders^a^	(Q81–82)
	Other syndromes	

*Note:* ICDv10-RCPCH = International Classification of Disease v10 (ICD10) code with British Pediatric Association (Royal College of Pediatrics and Child Health, RCPCH) one-digit extension.

a Minor anomalies. Codes for minor anomalies are not supplied and do not have a recording function in the app. Congenital skin disorders category includes advice on how to describe the anomalies and lists a number of minor skin anomalies, which need not be reported far surveillance purposes.

## Data Availability

Data sharing not applicable to this article as no datasets were generated or analysed during the current study.
